# Small RNA Pathways That Protect the Somatic Genome

**DOI:** 10.3390/ijms18050912

**Published:** 2017-04-26

**Authors:** Seogang Hyun

**Affiliations:** Department of Life Science, Chung-Ang University, Seoul 06974, Korea; sghyun@cau.ac.kr; Tel.: +82-2-820-5805; Fax: +82-2-825-5206

**Keywords:** transposable element, retrotransposon, PIWI, piRNA, endo-siRNA, genome stability, *Drosophila*

## Abstract

Transposable elements (TEs) are DNA elements that can change their position within the genome, with the potential to create mutations and destabilize the genome. As such, special molecular systems have been adopted in animals to control TE activity in order to protect the genome. PIWI proteins, in collaboration with PIWI-interacting RNAs (piRNAs), are well known to play a critical role in silencing germline TEs. Although initially thought to be germline-specific, the role of PIWI–piRNA pathways in controlling TEs in somatic cells has recently begun to be explored in various organisms, together with the role of endogenous small interfering RNAs (endo-siRNAs). This review summarizes recent results suggesting that these small RNA pathways have been critically implicated in the silencing of somatic TEs underlying various physiological traits, with a special focus on the *Drosophila* model organism.

## 1. Transposable Elements

**Figure 1:**
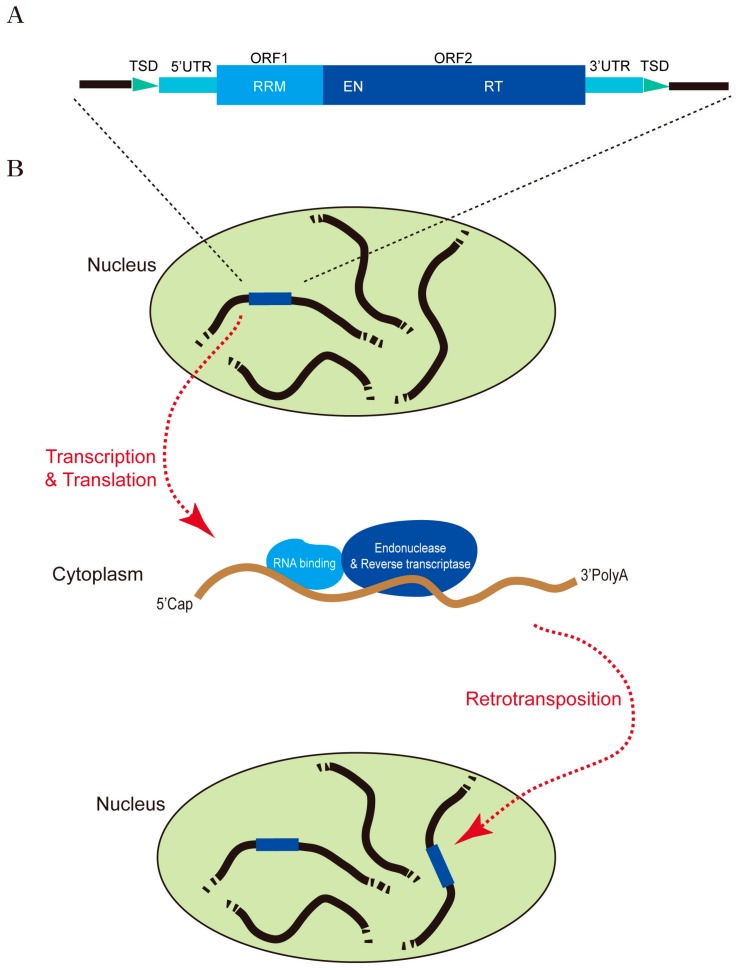
An example of a retrotransposon and its mobilization. (**A**) Genomic structure of the long interspersed nuclear element 1 (LINE1) retrotransposon. Full-length LINE1s are ~6 kb long and consist of a 5′ UTR, two open reading frames (ORF1 and ORF2), and a 3′ UTR flanked by target site duplication (TSD) sequences generated by previous retrotransposition events. The protein encoded by ORF1 has an RNA recognition motif (RRM), and the protein encoded by ORF2 has an endonuclease (EN) and reverse transcriptase (RT) domain. (**B**) Retrotransposition of LINE1. LINE1s are transcribed by RNA Pol II in the nucleus and their transcripts are exported to the cytoplasm. The proteins encoded by ORF1 and ORF2 are generated from the transcripts by translation, and these proteins act on the same transcripts from which they are translated, forming a ribonucleoprotein (RNP) complex. This complex moves into the nucleus, and acts at other loci of the genome, where a new copy of the DNA element, reverse-transcribed from the LINE1 transcript, is integrated.

Transposable elements (TEs) are discrete genetic elements that can move within the genome. Although TEs were first discovered in maize by Barbara McClintock in the 1940s [1], they were largely ignored for more than 30 years until they were found in a broad range of species. Since then, various TEs and TE-like sequences have been identified in many species, and the presence of mobile DNA elements in eukaryotic organisms is now widely accepted. Genome sequencing projects have revealed that TEs and their remnants occupy as much as 45% of the human genome [2] and 15–22% of the genome of *Drosophila melanogaster* [3,4]. TEs can be divided into two major classes, Class I and II, according to whether or not their transposition requires an RNA intermediate [5]. Class II elements consist of DNA transposons with inverted terminal repeats and direct repeats. Class II elements use a “cut and paste” process to excise themselves from the genome and insert themselves into a new genomic site without increasing the copy number. However, DNA transposons are generally inactive in humans and rodents. In contrast, retrotransposons, which constitute Class I elements, remain active in humans and rodents. These elements mobilize through a “copy and paste” mechanism of retaining the original copy and integrating a new copy at a new genomic location using RNA transcripts as transposition intermediates. Class I elements can be segregated into elements that are bounded by long terminal repeats (LTR), similar to those of retroviruses, and those that are not (non-LTR). Within the non-LTR class of retrotransposons, long interspersed nuclear elements (LINEs) and short interspersed nuclear elements (SINEs) remain active in humans and rodents. LINE1s in the human genome are approximately 6 kb DNA elements that encode open reading frames for the proteins L1ORF1p, an RNA-binding protein; and L1ORF2p, which is a protein with endonuclease and reverse transcriptase activity [6] (Figure 1).

Retrotransposons can induce genomic instability in various ways. One straightforward way is mutagenesis by insertion, which can induce genome disorganization and impact nearby gene expression. DNA double strand breaks that occur during retrotransposition contribute to genomic instability, and are highly mutagenic and susceptible to recombination [7,8,9]. The “copy and paste” mechanism of retrotransposons results in an increased copy number of retrotransposons in the genome, which has a substantial impact on the genome by producing insertion-mediated deletions and ectopic recombination [10,11]. In this way, active retrotransposons may become a source of endogenous mutagenesis that may underlie pathogenesis of many genetic diseases such as cancer and neuronal disorders. Furthermore, recent evidence indicates that as animals age or encounter stressful conditions, expression of retrotransposons is generally increased, which suggests that activation of retrotransposons may also contribute to the age-associated decline in organismal functions [12,13,14,15,16,17] (see below).

## 2. piRNA Pathways

Because TEs can serve as potent mutagenic factors contributing to genomic instability, organisms have adopted diverse molecular mechanisms to protect their genomes against TE activity. Among these, the most important one in animals is PIWI-interacting RNA (piRNA)-mediated TE silencing, a mechanism initially and most thoroughly studied in *Drosophila* [18]. piRNAs are a class of small RNAs, typically 23–30 nt long, bound by the PIWI subfamily of Argonaute (Ago) proteins. Piwi, a member of the PIWI protein subfamily, was originally identified in *Drosophila*, where it functions in germline stem cell maintenance and self-renewal [19,20]. Characterization of piRNAs in mammalian germline cells subsequently revealed that a previously recognized class of small non-coding RNAs in *Drosophila* called repeat-associated small interfering RNAs (rasiRNAs) corresponds to mammalian piRNAs [21,22,23,24,25,26,27,28,29]. PIWI proteins and piRNAs are highly expressed in germline cells, and their mutations lead to sterile phenotypes [30,31,32]. These phenotypes are believed to be due to defects in TE silencing, as mutations in the genes encoding PIWIs result in a dramatic increase in TE mRNA expression [33]. Due to their germline-specific characteristics, PIWI–piRNA pathways have been extensively investigated in germline cells, especially in the ovary of *Drosophila*. Sequencing of piRNAs in *Drosophila* germline cells revealed that they are mainly derived from TEs and TE-related genomic elements [25,26,27,34]. While microRNAs (miRNAs) are processed by Dicer from double-stranded precursors [35], piRNAs are produced in a Dicer-independent manner from single-stranded precursors [29]. Mapping of piRNAs onto the *Drosophila* genome revealed that dozens of genomic regions could be classified as piRNA clusters, which contain TEs and TE remnants [27]. These clusters are predominantly located in the pericentromeric and subtelomeric heterochromatin regions, and serve as templates for transcription of piRNA precursors. Other sources of piRNAs, such as the 3′ UTRs of protein-coding TE genes and dispersed euchromatic copies of TEs, have also been reported [27,36,37].

The *Drosophila* ovary consists of two distinct cellular compartments: germ cells and follicle cells. The latter are somatic cells that surround and support the developing germ cells [38,39]. piRNA pathways in the germ cells and follicle cells are thought to be distinct mainly due to different expression patterns of the three PIWI proteins Piwi, Aubergine (Aub) and Argonaute 3 (Ago3). Aub and Ago3 are predominantly expressed in the nuage, a perinuclear germline-specific structure, while Piwi is mainly localized in the nuclei of both germ cells and follicle cells [27,34,40,41]. These observations indicate that PIWI proteins have a distinct and discrete role in the biogenesis and mechanism of action of piRNAs, and that germ cells and somatic follicle cells may have different piRNA pathways. Investigation of piRNA clusters revealed that they could be transcribed either uni- or bidirectionally [27]. Most of these clusters are actively transcribed in germ cells, whereas only a few clusters—including the *flamenco* cluster—are active in somatic follicle cells. It is generally known that germline clusters have two promoters on each side, resulting in bidirectional transcription, however, *flamenco* is unidirectionally transcribed [33].

**Figure 2:**
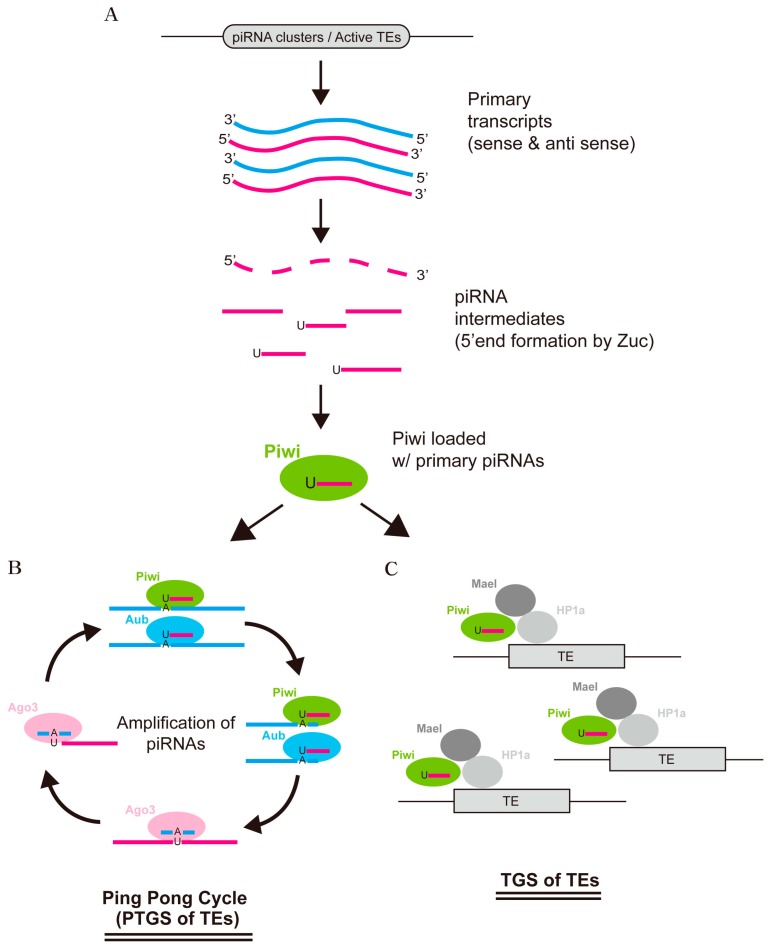
A model for piRNA biogenesis and its implications in TE silencing in the *Drosophila* ovary. (**A**) piRNA clusters or dispersed euchromatic TE regions are transcribed uni- or bidirectionally, generating primary piRNA transcripts with only sense or with both sense and antisense sequences, depending on the cell type (follicle vs. germ cells). After these primary transcripts are processed by yet unknown mechanisms, piRNA intermediates are cleaved by Zuccini, resulting in 5′ end formation. After this, transcripts with uridine (U) at the 5′ end position are preferentially loaded into Piwi, followed by trimming of the 3′ end to be matured with lengths of 23–30 nt. (**B**) In the germ cells of the ovary, the piRNAs generated in step (**A**) are loaded into either Piwi or Aub, and are engaged in the ping-pong cycle to be amplified in response to transcription of TEs, leading to post-transcriptional silencing of TE expression. (**C**) Piwi loaded with piRNA in the nucleus recognizes the active TE loci in the genome, where it recruits several heterochromatin-associated proteins, including HP1a and Maelstrom (Mael), ultimately leading to transcriptional silencing of TE expression. TGS, transcriptional gene silencing; PTGS, post-transcriptional gene silencing.

The current model of ovary piRNA pathways (Figure 2) illustrates that following transcription of a cluster, the transcripts are exported to the cytoplasm where they are processed into primary piRNAs and are loaded into Piwi. During this process, UAP56, a putative helicase, delivers the cluster transcript through the nuclear pore to the nuage where Vasa prepares the RNAs to be processed [42]. PIWI proteins with ribonuclease activity, such as Piwi, Aub, and Ago3, are believed to play a central role in the processing step [27]. The finding that Piwi-bound piRNAs exhibit a strong bias for uridine (U) at the 5′ end has led to a model of primary piRNA biogenesis where the 5′ end of the piRNA is processed first, followed by preferential loading of these piRNA intermediates with a 5′ U into Piwi, after which the 3′ end is processed by 3′ trimming activity [27]. Although the mechanism responsible for both 5’ and 3′ ends formation of piRNA remains incompletely understood, recent reports strongly indicate that the Zucchini (Zuc) endoribonuclease protein may act in generating the uridine biased 5′ end of piRNAs [43,44]. Recent studies further suggest that Zuc may also play a role in determining 3′ end of piRNA, generating phasing patterns of piRNAs bound by Aub and Piwi [45,46]. Moreover, Nibbler, a 3′ to 5′ exoribonuclease, has been shown to participate in shaping the 3′ end of piRNAs in a separate manner from that of Zuc [47]. Lastly, several other proteins, such as Armitage (Armi), Shutdown (Shu), and Vreteno (Vre), have been characterized that play a role in the correct loading of piRNAs into PIWI proteins [48,49,50,51,52] (Figure 2A).

In the germ cells, primary piRNAs generated by the pathway described above are believed to be amplified to produce secondary piRNAs through a process known as the ping-pong cycle [27,34] (Figure 2B). In the nuage, Aub or Piwi bound to a cluster-derived piRNA recognize an active TE transcript and cleave it, generating the 5′ end of a new sense piRNA, which is loaded into Ago3. Ago3 loaded with sense piRNA can recognize and cleave cluster transcripts, generating a new antisense piRNA bound to Aub or Piwi, thereby completing the cycle. This ping-pong cycle—with the cleaving activity of Aub and Ago3—can act as a self-sustaining mechanism for post-transcriptional gene silencing by cleaving and degrading the mRNAs transcribed from TEs. By using the cleaved products to make more piRNAs, this cycle could amplify its response to active transcription of TEs [27,34]. In somatic follicle cells, however, amplification of piRNA through the ping-pong cycle does not occur, and Piwi loaded with primary piRNAs is thought to move into the nucleus where it induces transcriptional gene silencing of TEs through chromatin modification (Figure 2C) (see below).

## 3. Role of piRNA Pathways in Somatic Tissues

Although the role of the piRNA pathways has generally been thought to be restricted to germline tissues, accumulating evidence in diverse organisms indicates that these pathways may also be present in various other cell types, ranging from pluripotent stem cells to differentiated somatic cells [53]. As discussed above, the functions of the Piwi and piRNA pathways have been characterized in somatic cells within the *Drosophila* ovary (ovarian follicle cells). Outside the gonads, Piwi is also found to bind to polytene chromosomes within salivary glands [54], and functions of PIWI proteins in the head of *Drosophila* were recently identified; Ago3 and Aub, which were thought to be germline-specific, were found to be expressed in distinct regions of the brain, where mutations caused the increased expression of several TEs [55]. Furthermore, previous genetic studies on the *Drosophila* eye color system implicated Piwi in position effect variegation, a phenomenon where genes situated near the heterochromatic region are expressed in a mosaic pattern in the tissues [26,54]. Functions of PIWI proteins have also been suggested in lower eukaryotes. *piwi* genes were found to be expressed in planarian totipotent stem cells, and knockdown of *piwi* gene expression was shown to cause the failure of body-part regeneration, leading to death [56].

Interestingly, a large number of studies found the ectopic expression of PIWI proteins in several types of cancer. PIWI proteins were reported to be overexpressed in seminoma, a testicular germ cell tumor [57], and PIWI expression was observed in a variety of somatic cancers such as gastric cancer, breast cancer, colon cancer, gastrointestinal stromal tumor, and renal cell carcinoma [58,59,60]. Furthermore, recent studies suggest that Piwi contributes to the growth of genetically induced malignant tumors in *Drosophila* [61,62]. In line with these findings, it is worth noting that genomic regions of LINE1s are hypomethylated in mammalian tumors and LINE1s can be reactivated in the pathological process of cancer [17]; this raises the possibility that activation of piRNA pathways may be a response to retrotransposon activation in cancer progression. Much investigation is currently underway to determine whether ectopic expression of PIWI proteins could have a causative role, and whether it could serve as a prognostic biomarker, in the development of various cancers.

Despite a number of cases showing the expression and functions of PIWI proteins in somatic tissues, current evidence supporting the existence of piRNAs in relation to the function of PIWI proteins in somatic tissues outside of the gonad is scarce. However, recent studies have begun to show that piRNAs are indeed functionally expressed in various types of the soma. Yan et al. showed the expression of small piRNA-like RNAs in various somatic tissues from mouse and macaque, using the analysis of high-throughput small RNA sequencing data and in situ RNA hybridization [63]. Eric Kandel’s lab provided evidence for existence of piRNAs and expression of PIWI proteins in neuronal tissues in both Aplysia and mouse, suggesting their roles in epigenetic regulation of memory-related gene expression [64,65]. Lee et al. also identified the expression of piRNAs in mouse brain tissues [66]. A very recent study has provided strong evidence for the presence of functional somatic piRNAs and PIWI proteins in *Drosophila* adult adipose tissues, showing that these piRNA pathways actively suppress TE mobilization to prevent metabolic dysregulation and lifespan shortening [67]. Further work will help to determine whether and how the protein components of piRNA pathways in somatic tissues outside of the gonad may work in concert with piRNAs.

## 4. Transcriptional Silencing of TEs by piRNA Pathways

Since ovarian follicle cells do not have a ping-pong cycle, and only Piwi among PIWI proteins is expressed in these cells, as well as its nuclear localization, it has been suggested that Piwi loaded with primary piRNAs may suppress the expression of TEs at the transcriptional level in the nucleus [33,53]. Indeed, several studies showed the changes in histone marks in many TEs after disrupting piRNA pathways using methods such as Piwi knockdown [68,69,70]. Use of the OSS/OSC cell line derived from the follicle cells of the *Drosophila* ovary has enabled further investigation of mechanisms of action of Piwi in transcriptional gene silencing. A recent study using this cell line showed that upon Piwi knockdown, RNA pol2 occupancy and H3K9me3 marks on TE genomic regions increased and decreased respectively, and that the formation of an H3K9me3 island on dispersed euchromatic TEs was dependent on Piwi and on transcription of the locus [71]. Moreover, Maelstrom (Mael), a protein previously known to be involved in germline piRNA pathways, was identified as a player in the downstream nuclear action of Piwi in transcriptional silencing of TEs [71,72,73]. Other studies suggest that Heterochromatic Protein 1a (HP1a), which binds to H3K9 methyl groups, plays a role in this silencing mechanism by showing its physical interaction with Piwi and derepression of TEs upon HP1a depletion [54,70]. A recent study also shows that Piwi physically interacts with histone H1, thereby increasing H1 density and decreasing chromatin accessibility at a subset of TE loci [74]. Based on these findings, a current working model proposes that Piwi loaded with piRNAs localizes to the transcription site of TEs by recognizing nascent TE transcripts, which recruits histone methyltransferases such as Su(var)3–9 and histone H1; methylation of H3K9 by Su(var)3–9 allows the binding of HP1a to the modified histones, inducing transcriptional silencing of TEs in collaboration with Mael and aggregated H1 (Figure 2C). It is clear that more factors may play a role in the epigenetic regulation of TE suppression initiated by Piwi, and further investigation is needed to fully understand this process. It is important to note that the mechanism of action of nuclear Piwi described above is postulated based on results from follicle cells of ovarian soma; future work is therefore warranted to determine whether other somatic tissues display similar mechanisms of transcriptional TE silencing by piRNA pathways.

## 5. Endo-siRNA Pathways and TE Silencing

**Figure 3:**
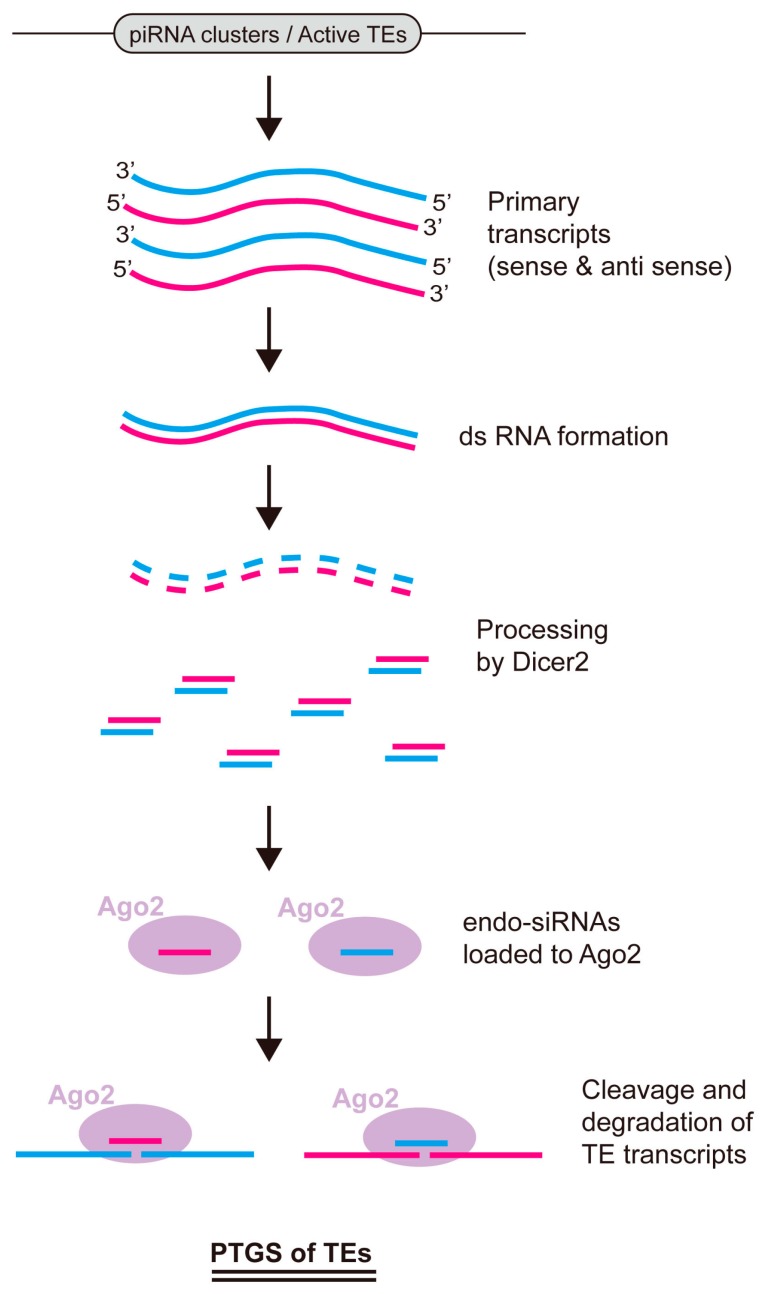
The endo-siRNA pathway in *Drosophila*. Endo-siRNAs arise from similar genomic regions to piRNAs, including piRNA clusters or active TE loci in euchromatic regions. Double-stranded RNAs (dsRNAs) formed from the primary transcripts of complementary sense and antisense sequences serve as the substrate for Dicer2, generating dsRNA intermediates with lengths of ~21 nt. One single-stranded RNA from these dsRNA intermediates is loaded into Ago2, executing cleavage and degradation of TE transcripts with complementary sequences. PTGS, post-transcriptional gene silencing.

Along with piRNAs, endogenous small interfering RNAs (endo-siRNAs) have also been found to suppress TE expression in both gonadal and non-gonadal tissues [75,76]. Long double-stranded RNAs formed from complementary TE transcripts have been shown to be converted into ~21 nt small RNAs that exhibit distinct characteristics from those of piRNAs. In *Drosophila*, these small RNAs specifically bind to Ago2, the effector protein of the RNA interference (RNAi) pathway (Figure 3). Sequencing of small RNAs in embryos, ovaries, S2 cell lines, and heads of *Drosophila* has revealed that endo-siRNAs mainly map across the region of TEs, heterochromatin, and intergenic elements within the genome, and sometimes arise due to convergently transcribed regions of two adjacent protein coding genes [75,76,77,78]. Endo-siRNAs do not show specific biased orientation of sense or antisense sequences relative to TEs, nor do they exhibit an obvious nucleotide preference at the 5′ end or any other specific position. However, they show a characteristic phasing pattern when mapped on the genome, indicating that Dicer2, the one of two *Drosophila* Dicer proteins implicated mainly in the RNAi pathway, may be involved in their process pathway. Indeed, mutation of *dicer2* was shown to eliminate the generation of endo-siRNAs in vivo [75,76,78].

The endo-siRNA pathway has been shown to play a role in TE silencing in mouse germline tissues; mutation of the pathway causes mouse infertility [79,80,81]. However, it seems that the germline-specific effects of endo-siRNAs in *Drosophila* are milder than in mouse, since smaller subsets of TEs are affected and flies were shown to be viable and fertile when *dicer2* or *ago2* was mutated [75,76,77]. It is possible that piRNA pathways are more redundant in silencing TE expression in *Drosophila* germline cells than in mouse cells. In line with this, Ago2-associated endo-siRNAs frequently map to piRNA clusters, indicating that both endo-siRNAs and piRNAs can be produced from the same primary transcripts [75,76,77,78] (Figure 3). Ago2 loaded with endo-siRNA is believed to induce the cleavage of TE transcripts complementary to endo-siRNA sequences, thereby degrading the transcripts and suppressing TE expression. However, increasing evidence raises the possibility that Ago2 loaded with endo-siRNAs is able to work in the nucleus as an inducer of transcriptional gene silencing via heterochromatin formation, like Piwi loaded with piRNAs [82,83,84].

Many studies in *Drosophila* have provided evidence for the roles of endo-siRNA pathways in silencing TE in fully differentiated somatic tissues. Ghildiyal et al. observed that ~21 nt small RNAs without obvious uridine bias at the 5′ end position were expressed in the head of *Drosophila*, a significant portion of which mapped to TE sequences [77]. The authors found that a subset of TE mRNA expression was upregulated in the head of *dicer2* or *ago2* mutants with decreased levels of endo-siRNAs. As briefly mentioned in the earlier section describing TEs, numerous studies in several organisms have suggested that age-associated loss of repressive heterochromatin structure leads to increased expression of corresponding TEs [85]. Several recent studies on *Drosophila* have provided evidence that activation of TEs in brain and adipose tissues contributes to age-associated decline in organismal functions, which could be modulated by manipulating the activity of Ago2 or Dicer2 [14,86,87]. Furthermore, a recent study in a *Drosophila* model of amyotrophic lateral sclerosis (ALS) has shown that siRNA-mediated silencing effects were compromised, and that the expression levels of endo-siRNAs and their corresponding retrotransposons were altered upon ectopic expression of human TDP-43, the ALS-causing protein [88].

## 6. Concluding Remarks

The piRNA and endo-siRNA pathways are now known to act as conserved surveillance mechanisms to suppress the activity of TEs in various types of animal cells, and are not restricted to germline cells. Although these pathways were identified nearly a decade ago, many questions remain unanswered regarding the biogenesis of these small RNAs, the mechanisms of their action, and their biological significance in normal and pathological contexts. What makes the activated piRNA clusters in germline cells different from those in somatic follicle cells? How can single-stranded transcripts of primary piRNA precursors be initially cleaved into piRNA intermediates and then loaded into Piwi? How can double stranded RNAs be made from TE or piRNA cluster regions to provide a substrate for Dicer to produce endo-siRNA? To what extent do piRNA and siRNA pathways participate in the process of organismal aging and various pathological conditions by protecting the genome from TEs? Furthermore, despite some evidence showing the functions of PIWI proteins in various somatic tissues, it is still unclear whether these functions are related to the expression of piRNAs, because reports supporting the existence of piRNAs in somatic tissues outside of the gonad have been scarce. Our understanding of the mechanism of action of nuclear Piwi and Ago2—which are loaded with piRNAs and endo-siRNAs, respectively—in the epigenetic regulation of TE silencing also remains fragmented. Regarding these issues, a recent report of crystal structure of Piwi loaded with piRNAs will facilitate our understanding of Piwi function in piRNA biogenesis and TE silencing [89]. The development of new biochemical methods, such as the establishment of a cell-free in vitro system [90], will allow further characterization of the piRNA and endo-siRNA pathways and their mechanisms of action. With the advances of next generation sequencing technology, as well as the application of genome editing using the CRISPR-Cas9 system in model organisms including *Drosophila*, future work will shed more light on the mysteries of these small RNAs.
